# The Impact of Insider Researcher Trainees in Recruiting and Retaining Latinx in an Outdoor Health Promotion Research Study

**DOI:** 10.1007/s40615-023-01642-1

**Published:** 2023-06-06

**Authors:** Rebecca Mendez, Edgar Velazquez, Alyssa Gimenez, Midley Michaud, Jaqueline Mendez, Miriam Wong, James Quesada, Leticia Márquez-Magaña, Cathy Samayoa

**Affiliations:** 1 Department of Biology, San Francisco State University, Hensill Hall 665, 1600 Holloway Ave, San Francisco, CA 94132 USA; 2 Internal Medicine, University of California San Francisco, San Francisco, CA 94110 USA; 3 The Latina Center, 3701 Barrett Ave, Richmond, CA 94805 USA; 4 Department of Anthropology, San Francisco State University, 1600 Holloway Ave, San Francisco, CA 94132 USA

**Keywords:** Community-engaged research (CEnR), Underserved populations, Recruitment and retention, Physical activity intervention

## Abstract

Latinx represent the second largest ethnic group in the USA and remain significantly underrepresented in research studies. Efforts to better include Latinx make use of community-engaged research (CEnR) approaches, peer-navigators, and cultural humility training for research teams. While these efforts have led to slight increases in Latinx participation, studies to identify strategic practices for better inclusion of Latinx participants are needed. The objective of this study was to qualitatively examine factors leading to successful recruitment and retention of Latinx participants in the Promoting Activity and Stress Reduction in the Outdoors (PASITO) intervention. For this intervention, 99 low-income Latinx clients in a local community were contacted and 52 participants were recruited (53%). All were retained in the 3-month intervention. Of these, 12 were interviewed within 6 months of the close of PASITO by bi-cultural and bi-lingual non-research staff. They conducted one-on-one structured telephone interviews. Of the twelve participants, three (25%) were men, nine (75%) were women, and the mean age was 43.7 (SD = 8.7). Four critical themes for the recruitment and retention of Latinx populations emerged from the interviews: (1) importance of insider researchers; (2) sense of community and belonging; (3) responsive programming; and (4) health-promoting activities. These findings support the significant role insider researchers can play, and social identity theory provides a useful framework for understanding the role of insider researchers in recruiting and retaining Latinx, and likely other minoritized groups, in clinical studies. Insider researchers possess the skills, training, community cultural wealth, in-depth understanding of their communities, and structural competencies that position them to carry out more inclusive studies to address the needs of marginalized communities and advance science.

## Background

### Latinx in the USA and Underrepresentation in Health Disparities Research Studies

Latinx represent 18% of the US population, making them a significant ethnic group as they are the most prominent and one of the fastest-growing minoritized groups in the country. They also bear an excess burden of disease [1]. Obesity, diabetes, and cardiovascular disease are disproportionately prevalent among Latinx populations in the USA. Furthermore, Latinx groups face disparities in cancer-related health outcomes [2] and bore the greatest burden of COVID-19 deaths in California [3]. Despite the excess burden of death and disease, Latinx are underrepresented in clinical and biomedical research; fewer than 5% of human subject participants are Latinx [4]. The lack of equitable representation of minoritized populations, including Latinx, in biomedical research studies contributes to health disparities as it limits scientific rigor and impact that could benefit all populations [5–7].

### Barriers to Equitable Representation of Latinx

Despite the 1993 National Institutes of Health Revitalization Act, which called for the inclusion of women and minorities in research, dominant majority researchers have been largely unable to meet this national call for inclusion [4]. Dominant majority (outsider) researchers often report significant challenges to recruiting and retaining women and minority participants and ultimately fail to adequately represent Latinx groups in clinical studies. In fact, Latinx have been referred to as a “hard to reach population” [5]. However, studies examining barriers to participation in clinical studies report that Latinx communities are willing to enroll if given trustworthy and accessible opportunities to participate [8]. Meeting these reasonable criteria for participation has been shown to overcome reported barriers, which include historical, systemic, and logistical factors, to an adequate representation of Latinx groups in clinical studies [6]. A history of unethical treatment by scientists and medical providers has deemed research studies untrustworthy by ethnic minority populations and systemic, logistical, and social challenges to participate are infrequently recognized and even more rarely addressed [9]. For Latinx, a historically reverberating injustice that accounts for significant distrust is the State law that allowed the forced sterilization of over 20,000 Latinx women in California for more than 70 years until it was finally repealed in 1979 [10]. Additionally, systemic factors such as the lack of opportunities to participate in clinical research at sites where Latinx seek health services, English-only requirements, and the lack of culturally and linguistically relevant education regarding clinical studies limit participation [11–15]. Moreover, logistical issues such as lack of transportation to study sites, cost to participate due to additional expenses, and loss of income if taking time off of work [16] have been reported as significant barriers to Latinx representation. Finally, racial/ethnic biases in recruitment exist (e.g., belief that Latinx, and other racial/ethnic minority groups, are challenging and otherwise non-ideal participants), making participation inaccessible to Latinx because they are not even asked to participate by biased researchers [6, 17, 18]. Taken together, it is clear that traditional recruitment approaches are inadequate to carry out genuinely inclusive studies [5, 19].

### The Importance of Insider Researchers in Recruiting and Retaining Latinx in Health Disparities Research

Studies that aim to increase Latinx representation in research studies and address health disparities have moved outside of traditional academic settings and into communities. Community-engaged research (CEnR) approaches and the use of community health workers, lay outreach workers, patient navigators, and *promotores* in guiding and implementing research studies have significantly contributed to increasing the number of Latinx participants in research studies [20–22]. This has resulted in a greater understanding of minority health, and enabled the advancement of health disparities research [20, 23]. Taken together, these innovative research approaches expand the tools for how research is conducted, leading to more inclusive studies that improve the rigor of the results obtained and their promise for improving health [24]. While these approaches have focused on increasing community participation across the engagement continuum for research and have included members of the community on the research team (e.g., community health workers, *promotores*), the role these insider researchers can play in improving inclusion of minoritized groups in clinical studies has yet to be fully investigated and is an area in need of further research.

A growing body of evidence indicates that racial/ethnic concordance between study staff and participants may have an effect on recruitment and retention. A meta-analysis of clinical studies by Yancey et al. [25] demonstrated that inclusion of racial/ethnic minorities on research teams improved recruitment and retention of minority research participants [25]. Thus, the inclusion of ethnic minority research staff was found to be an effective facilitator of inclusive study participation. This effectiveness can be attributed to implicit trust of communities for which ethnic minority staff are culturally representative, their innate understanding of social and cultural norms, and oftentimes their deeply personal reasons for conducting the research that increases agency for the work [26]. These factors may also explain the finding that Latinx insider researchers (Latinx researchers working with Latinx participants) effectively recruit and retain Latinx in research studies [20, 27, 28]. These factors also align with the assets described for individuals who possess community cultural wealth [29]. The elevation of these assets and insider researchers who possess them are critical to mitigating the outcomes of justifiable distrust by communities of color terrorized by historical misconduct in medicine and scientific research [30–32].

### The Promoting Activity and Stress Reduction in the Outdoors (PASITO) Intervention

The PASITO study investigated the health impact of a group-based outdoor physical activity intervention for low-income racial and ethnic minority groups, with a focus on Latinx. This population has reported experiencing the highest levels of stress compared to other ethnic or racial groups [33]. To address this issue, the PASITO study measured both perceived and physiological stress. Previous research has found that 80% of Latinx have reported experiencing high levels of neighborhood stress, which can significantly impact their psychological and physical health [34]. Additionally, Latinx populations often live in neighborhoods with inadequate resources, such as a lack of green spaces, and higher levels of neighborhood stressors. Together, these factors contribute to health disparities in the Latinx population.

As part of the recruitment and retention strategy, the study team partnered with program providers (the East Bay Regional Park District and the San Francisco Recreation and Parks Department) as well as a trusted community organization (The Latina Center). Recognizing the significant distrust of research in Latinx communities, the PASITO study team strategically used insider researchers and were successful in recruiting and retaining Latinx study participants [35, 36]. Insider researchers were strategically included in all phases of the research continuum—from study conceptualization to the dissemination of research findings—to better include minoritized populations as research participants for the reduction of health disparities [37].

Insider researchers in the PASITO intervention underwent comprehensive training that incorporated best practices for engaging with study participants, drawing from both a community cultural wealth model and a structural competency conceptual framework [29, 38, 39]. The structural competency frameworks encompass a deep understanding of how various factors at multiple levels, such as social, economic, political, and cultural structures, influence community health outcomes. The ultimate objective of this training was to enable insider researchers to apply their lived experiences to effectively address health disparities through community-focused research.

Structural competency includes recognition of the social, economic, and political conditions that create unequal representation of Latinx in research studies and drive health inequities. Thus, insider researchers were specifically trained on how to leverage their personal lived experiences, deep understanding of the community, cultural capital, and structural competency, to bridge the gap between academic research and community well-being. For example, insider researchers had a keen understanding of the community assets such as parks that were easily accessible and safe, had deep understanding of the values that motivated the community to engage in a health-promoting activity, and were also aware of the potential barriers that could arise, such as challenges with childcare. As part of their training, inside researchers participated in webinars on social determinants of health, health disparities conferences, and monthly discussions during laboratory meetings centered on how multi-level factors impact health outcomes among marginalized communities. Through these training approaches, insider researchers recognize the impact that these factors have had in their own communities, and worked together to identify ways to leverage their community cultural wealth and structural competency for research with communities (Table 1).

**Table 1:** Demographic characteristics of PASITO participants who were interviewed for the qualitative study

Characteristics	Total (*n* = 12) (%)
Age (mean ± SD)	43.7 ± 8.7
Sex	
Male	3 (25)
Female	9 (75)
Employment status	
Full-time	5 (41.6)
Not currently employed	3 (25)
Other	3 (25)
Missing or did not disclose	1 (8.3)
Education	
Some high school or less	6 (50)
High school diploma or GED	1 (8.3)
Missing or did not disclose	5 (41.6)
Income	
< $25,000	5 (41.6)
$25,000–$49,999	1 (8.3)
$50,000–$74,999	1 (8.3)
Missing or did not disclose	5 (41.6)

Barriers to participant recruitment and retention, such as transportation and lack of childcare, were addressed by coordinating carpool and inviting family members to join the nature walks. Furthermore, educational outreach, recruitment, and programming were conducted in both Spanish and English, and insider researchers were strategically matched with racially or ethnically concordant participants to create a culturally relevant and inclusive research environment [35]. The demographic characteristics of the insider researchers are described in Table 2.

**Table 2:** Demographic characteristics of insider researchers in the PASITO study

Characteristics	Total (*n* = 8) *n* (%)
Age (mean ± SD)	27 ± 4.8
Gender	
Male	4 (50)
Female	4 (50)
Ethnicity	
Latinx	7 (87.5)
Other	1 (12.5)
Education	
Undergraduate student	5 (62.5)
Bachelor of Science	1 (12.5)
Master of Science	1 (12.5)
Ph.D. graduate	1 (12.5)
First generation-college	7 (87.5)
Country of family origin	1 (12.5)
Asian	1 (12.5)
South America	1 (12.5)
Central America (Guatemala, El Salvador)	1 (12.5)
Mexico	3 (37.5)
More than 1 (Mexico and Guatemala)	1 (12.5)
Income	
< $25,000	5 (62.5)
$25,000-$49,999	3 (37.5)

Fifty-two participants were recruited to the parent PASITO interventions. The average age was 42 years, the majority (63%) were female, and 66% reported being employed either full-time or part-time. Forty-three out of the 52 participants in the parent study were Latinx, and of those, 42 provided salivary samples and were included in the analysis. Findings from the parent study demonstrated that stress, both measured by the analysis of salivary cortisol and self-reported perceived stress, significantly decreased over the course of the event (*p* < 0.05) [36].

As Latinx health disparities continue to grow, it is imperative that we better understand how to overcome barriers to inclusion of minoritized populations in clinical studies. Therefore, this qualitative research study, conducted after the conclusion of the PASITO study, sought to identify factors responsible for successful recruitment and retention of Latinx. Specifically, unique information about the experiences and perspectives of Latinx participating in a study with Latinx insider researchers were obtained. This information is unique because few clinical studies are implemented by Latinx teams due to their significant underrepresentation in the biomedical research workforce [40]. This study sought to gain a greater understanding of Latinx participation in a clinical study with insider researchers and the impact that can have on the quality and rigor of research made possible by enhancing the diversity of the biomedical research workforce.

## Methods

### Recruitment

This study used in-depth interviews to comprehensively explore and gather information about the perspectives of Latinx living in Richmond, California, related to participating and remaining in the PASITO program.

At the conclusion of the parent PASITO study, informants were randomly recruited to participate in an interview to assess their experiences and perceptions in the study. Participants were contacted for interviews, until the data saturation point was reached, an essential indicator that the sample was adequate for our study [41]. A total of thirteen participants were contacted, and 12 completed the full interview. Informed consent was obtained verbally during the scheduling process and then was verified verbally at the beginning of the interview session. All participants responded, but one became unavailable (92% participation rate). Interviews were conducted by telephone in the month of April 2018 (within 6 months of the last PASITO study visit) and ranged from 20 to 30 min long. All informant interview discussions were facilitated, audio-recorded, and transcribed verbatim in Spanish and translated to English by a consultant proficient with the Spanish and English languages.

### Demographics Characteristics

Demographic measures were collected during enrollment of the parent study and included the following self-reported measures: age, gender, employment status, education level, and income level.

### Interview Tool/Instrument

The interview tool guide consisted of six open-ended questions designed to capture participants’ experiences and their perceptions about joining and remaining in PASITO. For example, the interviews began with the following general question to assess factors influencing participants’ decisional process: “How did you decide to join and participate in a clinical trial like PASITO?” The remaining 5 questions were designed to evaluate motivating factors for remaining in the study and the likelihood of participation in other studies that included insider researchers (see the Appendix).

### Data Analysis

After translation from Spanish to English, transcripts were independently coded by three researchers who had been previously familiarizing themselves with all transcribed interviews. This first round of coding led to the development of the codebook. It was used by the researchers to discuss and attain consensus with each round of coding. Data were subjected to inductive thematic analysis, which permitted the investigators to assess the data and identify patterns, concepts, and ideas. To facilitate the production of high-quality findings, a categorization process by Consta [43] was used to organize data into groups of interconnected codes and use the theme development method described in Vaismoradi et al. [42, 43]. A conceptual model was created to explain relationships among main themes using a Grounded Theory approach after recurrent peer debriefing sessions (see Fig. 2) [44]. These themes were linked back to the key research question (Why did participants join and remain in the PASITO study?) and relevant literature. Codes were quantified to determine percent coverage within themes and representativeness across interviews. Theme saturation was identified by the sixth interview through a comparative analysis. While interpretation was not verified with interviewees, credibility was obtained by applying theory-grounded interpretation and accepted qualitative methods.

## Results

### Sample Characteristics

**Fig. 1:**
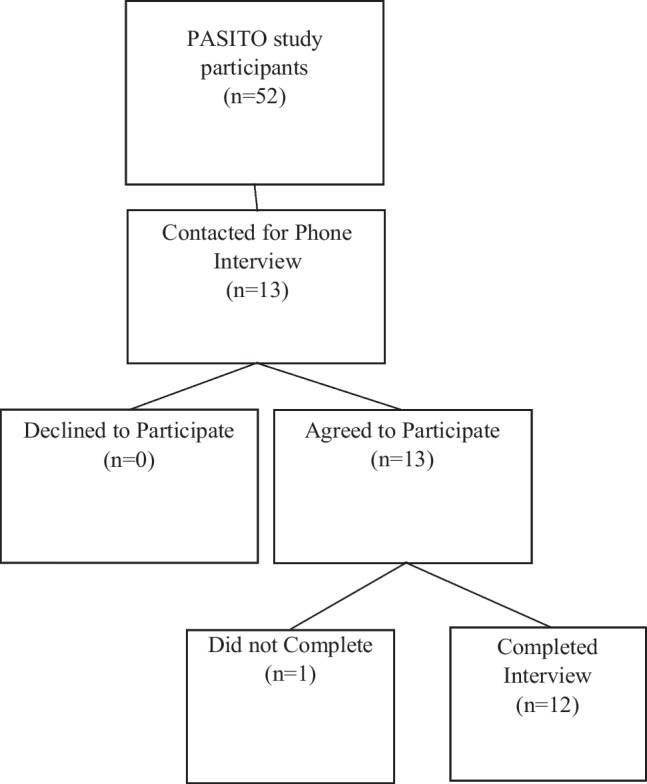
Flow chart of qualitative study participants recruited from the PASITO project, Richmond, CA, 2017–2019

A total of 12 participants completed the phone interviews (Fig. 1). The mean age was 43.7 years (SD =  ± 8.7; range 30–58), and the majority (75%) were women (Table1). Five participants were employed full-time, and six had some high school education or less. Forty-one percent had an annual income below $25,000. Thus, the group of participants who were interviewed were primarily low-income Latinx often considered by researchers to be “hard to reach” in clinical studies [45].

### Participants’ Experiences and Perceptions

**Fig. 2:**
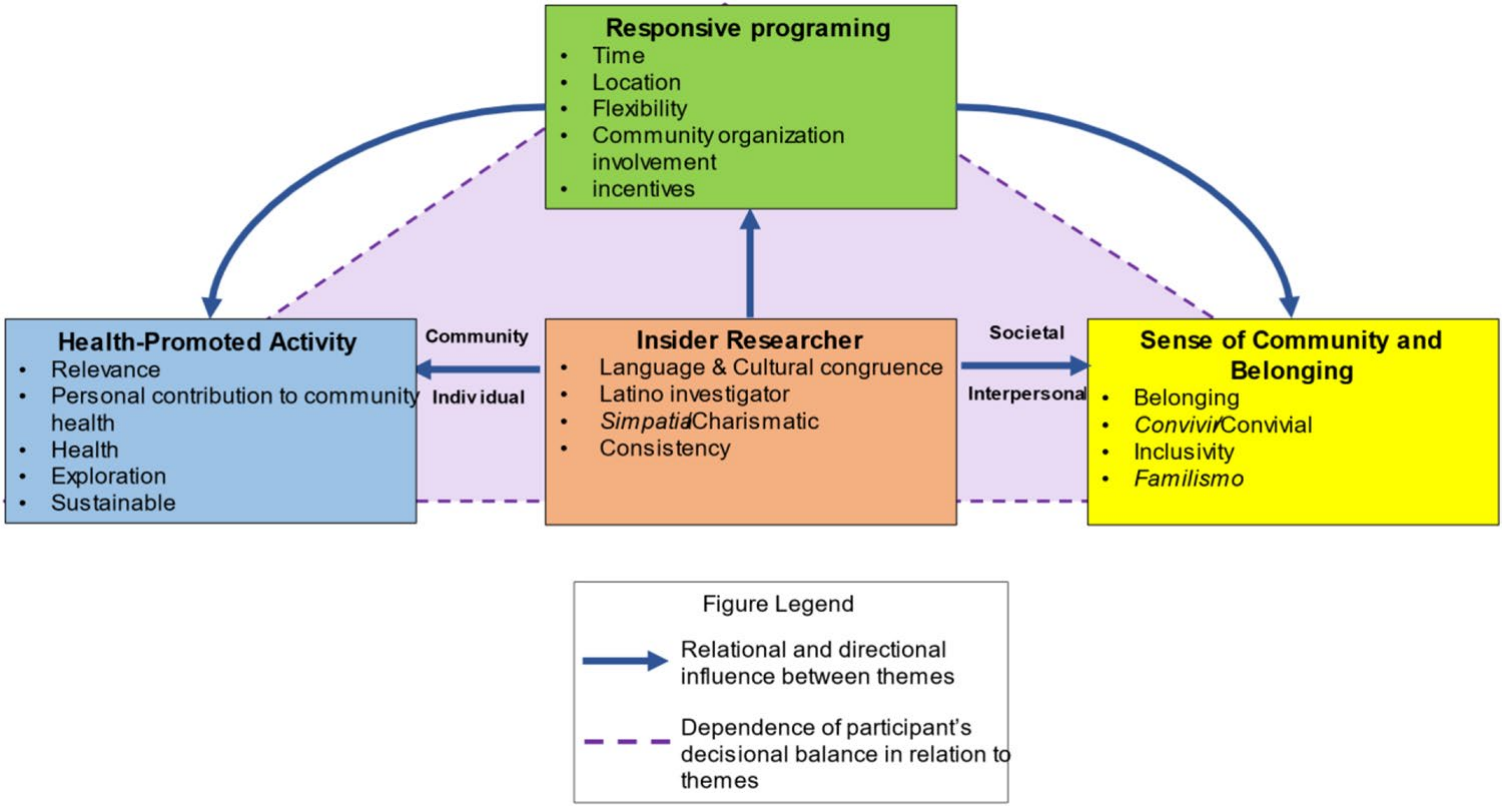
Theme conceptualization and categories

Four critical themes emerged from participants’ interviews: (1) strong effects of insider researchers; (2) a sense of community and belonging; (3) accessible programming; and (4) health-promoting activities. These key findings were identified as the main factors influencing participation of individuals joining and remaining in PASITO. Upon in-depth analysis of responses, a model depicting how major themes interact was created (Fig. 2). In the conceptual model (Fig. 2), the arrows depict the directionality of the relationships of each of the four themes. At the center, insider researchers were found to influence and connect each theme. In our study, participants perceived insider researchers as part of their community who spoke to and connected with members during the program, fostering a sense of community and belonging. Their cultural competency and experience facilitated the cultural knowledge and skills required to understand participants’ needs (e.g., health-promoting activities), thus reducing cultural and practical barriers to participation and increasing program access.

### Importance of Insider Researchers

Participants reported that effective recruitment and retention were due to the quality of interpersonal interactions reported between participants and culturally concordant study team members. This was indicated by the rapport and the level of trust participants reported about the insider researchers who implemented the PASITO intervention.

*We always felt like they were friendly and always thanking us for participating. They would come to our community and would get involved with all that we did, and I think that’s what made people stay in the study. To see them as not just doctors doing experiments, but seeing them as someone like us, of our community, working to improve the quality of life for Latinos*.

Interview responses also highlighted a perception by participants of the critical role the insider researchers played in keeping participants motivated in the study. For example, some participants perceived the researchers as giving “love” by showing a high level of commitment towards participants, the project, and the community:

*I would see them every time there [at the nature walks], it wasn’t like one day they were there and the next day they were gone. They were always present every time we had our nature walks and that gave the project a sense of seriousness. A sense of seriousness towards the project, and the love they gave in efforts to develop the project for the Latino community.*

Participant’s interviews also revealed key factors regarding motivation to participate in the study, and included clear communication using the community’s preferred language and messaging that displayed a nuanced understanding of cultural contexts. Insider researchers were commonly mentioned as the entities best suited to fulfill the latter role likely because of their established social and familial ties to the Latinx community:

*I identified [with the project] more since they [the research team] explained everything using the language we practically speak; there was a “Latino flavor.”*

In addition, insider researchers created an environment that made participants feel valued, comfortable, and respected. As a result, this led to participants wanting to support the professional development of the research team by remaining in the study.

*The team had a lot of energy for our community. And it is very rare to see young men like them, engaging respectfully with confidence and speaking of values. Even though they were young, they had a lot of wisdom and they made us feel comfortable. All their efforts were to support our community and I felt passionate to see that [the research team] was doing a project that focused on Latinos and participating was a way to support the students that were working on their project too. They are the future of this country and if we don’t support them who will? Right?*

### A Sense of Community and Belonging

Participants voiced that the PASITO study spurred feelings of belonging by welcoming family members and friends. Family members and friends were invited to participate in the nature walks in response to multiple requests by participants. Moreover, the inclusion of participants’ children helped to overcome a voiced barrier to participation (i.e., lack of childcare).

*Well, I really liked the fact that there were different people of different ages. They did not include just one age group, like, only participants of this age group will participate, but young people, adults, older adults could participate and that made the study more interesting because there were people of different ages involved…And it became something like a family. We were able to take our children, we were able to invite friends even if they were not part of the study, but they were always welcome and well received at the nature walks. This allowed us not to tell ourselves that we did not have anyone to take care of our children but instead made it easy for us to participate because our children were welcome to come with us, and the team made us feel that our children were always welcome.*

Participants described the nature walk environment as a communal activity and stressed the camaraderie between members, which shaped their experience in their study. For one respondent, this experience was defined as “conviviendo,” which in Spanish literally means “living together.” It is a term used to capture the feelings of living in a warm and supportive social environment.

I always thought that when we are involved in groups from the same community, that is another way to maintain a community [conviviendo]. Do you get me? Because in this country that tends to get lost. We, who come from other countries, tend to be isolated, and we don’t get opportunities to practice our cultures, and we are not able to cultivate our roots. Then, there comes a moment where we feel the need to be part of something or to be someone, or be part of a community. So that was a great reason for me for why I stayed in PASITO. In the end that’s what was accomplished—a small community.

### Responsive Programming

The ability of the research team to modify programming to make participation as accessible and convenient as possible was a factor that facilitated participation. Interviewees highlighted the convenient locations for walks, such as parks close to home, and that convenient times and transportation were offered.

*To tell you the truth it was very convenient for me because I did not have to travel far and I did not have to commit to a whole day, I just had to wake up early. It was only two to three hours and I would have the rest of the day free. And another of the reasons was that the hikes were not very long, and they did not take too much time, and it did not require a whole day, and I liked that…and that helped me in continuing my participation.*

In addition, interviewed participants widely acknowledged the flexibility of the research team to adapt programming as needed. For example, the team accounted for differences in walking ability levels and participant availability.

*I liked that there were people that walked very fast and others very slow, so they made two groups, the fast ones, and the slow ones. I liked that they would find alternatives to keep us motivated.*

Additionally, recruitment from a trusted community organization, the Latina Center, was perceived by interviewees as responsive to community needs. It was recognized that the research team worked with the Latina Center leadership to create a walking program and that it was made accessible by basing it at the Center.

*Because I work for Latina Center and they already had a walking program, and I think it was every month. And after I heard that it was a very dynamic project and they [the academic-community partnership] wanted to investigate the stress levels we were experiencing in the community, and they asked us if we wanted to participate. And I participated because it was with the same agency that they went to present the project.*

### Health-Promoting Activity

The overarching goal of PASITO to reduce the effects of chronic stress and promote health was a facilitator for participants’ decision to engage and remain in the study. In particular, the idea of healing in nature to reduce their stress levels appealed to most participants. Interviewees also expressed that a nature walk provided them with the opportunity to exercise to improve their health and the health of other members in their community.

*One of the reasons [I remained in the study] was to improve my health. To be in a place where you can breathe freely, in a place where it is not noisy and not stressful.*

*They [the researcher team] talked about a project that sounded interesting because it was to help our community and it was something we can be involved in… you know, in exercising. And because right now, we, Latinos are having a lot of problems related to stress, it may seem that we don’t care, but we do, and sometimes we do not know how to cope with it.*

Many of the participants expressed the benefits of repeated nature walks and their personal interest to explore nature deeply. The enthusiasm expressed for the nature walks indicates sustainability of the intervention after the PASITO study:

*When I finished the first hike, I felt so good that I said to myself, let’s see how the next one will be like. And every time I felt better and better.*

*What motivated me is that I love being in nature, I love going to parks… I love being in nature and it was a driving force and a challenge for me.*

And, finally, even though participants expressed appreciation for the financial incentive provided to participate, it was not a major driver for involvement in the study.

## Discussion

The objective of this qualitative study was to identify factors leading to successful recruitment and retention of Latinx participants in the Promoting Activity and Stress Reduction in the Outdoors (PASITO) intervention, a health promotion research study. Four critical themes emerged from the interviews: (1) importance of insider researchers; (2) sense of community and belonging; (3) health-promoting activities; and (4) responsive programming.

### Summary of Results

#### Importance of Insider Researchers

Through our qualitative inquiry with community members, we found that the inclusion of insider researchers in the PASITO study influenced the participation and retention of the recruits in the program (Fig. 2). We found that participants perceived insider researchers as primary representatives and that they were effective in establishing rapport, communicating in the participants’ native language, sharing their beliefs, and making them feel that their involvement was important and would result in significant contributions to the scientific field. Hence, all these qualities may have cultivated and fostered a sense of community and belonging among members and researchers. Their cultural competency and experience facilitated the cultural knowledge and skills required to understand participants’ needs (e.g., health-promoting activities), thus reducing cultural and practical barriers to participation and increasing program access. When asked about their willingness to engage in future studies led by insider researchers, all interviewees responded positively. They reported being willing to be part of studies that could help the insider researchers advance their careers and provide meaningful contributions to the field of minority health, nature-based interventions, and stress biology. Taken together, these results demonstrate that populations deemed to be “hard to reach” by others [5] are reachable by insider researchers. These findings are on par with several other studies showing that with cultural congruence (when researchers and participants share culture and/or language) communication is improved, misunderstandings are reduced, and both participants and investigators gain more confidence with interpersonal interactions [46–49]. This result addresses a prominent and cross-cutting challenge voiced by NIH leaders in 2015 to identify improvements in quality and outputs of research as a result of enhanced diversity of the biomedical research workforce [50].

#### Sense of Community and Belonging

A sense of community (SOC) was identified by participants as an influencing factor that persuaded participants to remain in the PASITO study. Prior studies have shown that SOC increases program participation [51, 52], increases the psychological empowerment of members of a group [53], and improves overall community health and well-being of community members [54, 55]. A comprehensive systemic review study conducted by George, Duran, and Norris [6] illustrated that Latinx, and other minority groups, were more likely to participate for altruistic reasons rooted in cultural and community priorities that arise to a sense of community [6]. Similarly, in our study, SOC facilitated the decisional balance by maintaining feelings of belonging, inclusivity, convivial environments, and altruism towards others in the program and future generations. Community members felt that they mattered to each other and the research group and relied on the belief that members’ needs would be met through their commitment to being together as previously supported by other studies [6, 56].

#### Responsive Programming

A 2018 study by Anderson et al. of over 12,000 individuals showed that participation in clinical research was oftentimes perceived as burdensome [57]. Minority communities, including Latinx, have been deemed as “hard to reach”; thus, understanding and addressing community barriers to participation are critical for inclusion studies. For Latinx, the concerns of participating in research studies have ranged from issues of mistrust, fear of discrimination, scheduling conflicts, lack of transportation, childcare, and other barriers [7, 16, 25, 58]. As a result, they may be reluctant or completely unwilling to participate [14, 59, 60]. In our study, respondents were willing to participate and remain in the study because they perceived fewer barriers to participate. The proximity of the study’s location (e.g., community center and parks), the ability to bring children to study visits, flexibility in participants’ abilities, and the selection of days and times that did not interfere with members’ work schedules were all mentioned in the interviews to be appropriate and manageable.

#### Health-Promoting Activities

Outdoor park interventions that can reduce the impact of stress may be particularly relevant and feasible among Latinx groups. In our inquiry with community members, participants showed a high level of appreciation for parks and natural settings; for many, nature was considered a social experience. They could embrace and maintain their cultural identity with one another and foster social cohesion in natural settings. These factors have been shown to reduce cortisol (stress hormone) and the harmful effects of chronic stress [61]. Participants in our study voiced an innate connection and attraction to nature and felt more self-assured about their life situations. Similarly, outdoor spaces and outdoor activities in nature have been shown to positively affect individual moods and behaviors, and increase social cohesion, as well as improve feelings of empowerment and confidence [62–65].

Social identity theory provides a useful framework for understanding the role of insider researchers in recruitment and retention of research participants, particularly in health equity and biomedical research studies. According to this theory, individuals define themselves based on their membership in social groups, which shapes their attitudes, beliefs, and behaviors [66]. Insider researchers, who share the same social identity as the communities being studied, are uniquely positioned to enhance recruitment and retention efforts by leveraging their insider knowledge, community cultural wealth, and cultural competence. This study demonstrated that insider researchers brought value to the study given their structural competencies, linguistic and navigational capital, and ability to build rapport with participants. They were identified as trusted community leaders, who also shared a social identity with participants, to promote the study and recruit participants who may have had apprehension and mistrust of research studies. Insider researchers who knew the history of the community and their concerns regarding the ethics of research may have provided a sense of belonging to participants, and in turn strengthened the social identify of participants in the group. Insider researchers also designed recruitment materials that were culturally relevant and demonstrated knowledge of the built environment of the community when selecting sites for the outdoor activity. Recruitment and retention strategies that honored the values of the participants, community leaders, and insider researchers, who all share a social identity, resulted in greater participant engagement, as evident through our interviews.

Moreover, insider researchers helped reduce mistrust and apprehension that minority populations may have had towards biomedical research due to a history of exploitation and lack of inclusion. By acknowledging the social identities of participants and leveraging the skills and knowledge of insider researchers, heath equity biomedical research studies can be designed and executed in a more culturally responsive and equitable manner. For example, offering incentives that align with the values or interests of the community, such as giving participants gift cards to local groceries stores, or providing resources or support that address specific barriers to participation, such as carpooling and allowing for children and extended family to participate in the outdoor activities, can improve retention rates. Social identity theory provides a framework for understanding the value that insider researchers bring to the recruitment and retention of diverse participants in research and can be utilized in future research studies aiming to recruit historically underrepresented participants.

### Limitations

This study has several limitations. First, these findings represent the perception of Latinx participants who were part of the PASITO project and resided in Richmond, CA, and, therefore, may not be generalizable to other communities. Second, our sample size of 12 participants was small which could impact the reliability of the findings. Furthermore, the interviews conducted were brief and designed to capture general perceptions regarding factors that influenced participation and retention. Comprehensive in-depth interviews that broaden the scope of questions with a larger sample size and that include communities historically underrepresented in research studies may provide insights into other factors that are important in the decision-making process of minorities to engage, enroll, and remain in research studies. And lastly, our study protocol did not include interviewing the insider researchers for their perspective and motivations which may have also added insight into the successful recruitment and retention of study participants.

## Conclusion

The underrepresentation of Latinx in research studies presents a challenge to achieving health equity [67]. In our study, four critical themes for the recruitment and retention of Latinx populations emerged from the interviews: (1) importance of insider researchers; (2) sense of community and belonging; (3) responsive programming; and (4) health-promoting activities. Central to these themes was the critical role that insider researchers played in facilitating cultural knowledge and reducing cultural and practical barriers to participation. These findings support the significant role of insider researchers in recruiting and retaining Latinx individuals, and likely other minoritized groups, in clinical studies. Insider researchers, in addition to being perceived by participants as primary representatives, speaking to and connecting with community members during the study, were also perceived as integral to the participants’ decision-making process regarding participation and retention in the study as well as being at the center of each of the other influencing factors and themes that were identified, including a sense of community and belonging, responsive programming, and health-promoting activities. Furthermore, social identity theory provides a framework for understanding the value that insider researchers bring to the recruitment and retention of diverse participants in research studies. The NIH’s continued efforts to invest in diversifying the biomedical workforce can result in a biomedical workforce that better represents the whole US population and its communities [40, 50]. Increasing the number of insider researchers could result in more diverse and equitable research studies. This study highlights how the inclusion of insider researcher trainees may better meet the research needs of the USA and be important in eliminating health disparities. Insider researchers possess the skills, training, and in-depth understanding of the community that position them to carry out studies that are inclusive and better address the needs of marginalized communities to advance science.

## Data Availability

Not applicable.

## Appendix. The Interview Tool Guide

English translation

Introduction:

Hello my name is ______and I work with the team from PASITO. I am calling to ask you a few questions regarding your experience and the reasons that motivated you to participate; and the reasons that kept your interest in the study. Would you give us permission to interview you? And do you give us your permission to record you? Remember this interview is completely confidential and your name and other personal information will be left out of the analysis and any publication.

Now I would like to ask the question again: Is it ok with you to record this interview?

Before we start, do you have any questions for me?

- Q1. Then I will start with the questions. The first question: How did you make the decision to join and participate in a clinical trial like PASITO?
- Q1a. What factors/motivators motivated you to participate in PASITOs?
- Q1b. And what kept you coming back throughout the three months of the study?
- Q1c. What qualities of the research team members, if any, motivated you to participate over the period of three months?
- Q2a. What else can you share about the reasons you participated in PASITO?
- Q2b. The last question: Are you more likely to participate if the research team is made up of student researchers?
- Q3. Is there anything else you would like to share?

Thank you, I will give all this information to the team and many thanks for your participation.
